# Incidental finding of *Clostridium perfringens* on human corpses used for the anatomy course

**DOI:** 10.1007/s12565-022-00699-y

**Published:** 2022-12-05

**Authors:** Alexander M. Kerner, Andrea J. Grisold, Freyja-Maria Smolle-Jüttner, Niels Hammer

**Affiliations:** 1 Division of Macroscopic and Clinical Anatomy, Gottfried Schatz Research Center, Medical University of Graz, Auenbruggerplatz 25, 8036 Graz, Austria; 2 D & R Institute of Hygiene, Microbiology and Environmental Medicine, Medical University of Graz, Neue Stiftingtalstrasse 6, 8010 Graz, Austria; 3 Division of Thoracic and Hyperbaric Surgery, Department of Surgery, Medical University of Graz, Auenbruggerplatz 29, 8036 Graz, Austria; 4 Department of Orthopedic and Trauma Surgery, University of Leipzig, Leipzig, Germany; 5 Division of Biomechatronics, Fraunhofer Institute for Forming Tools, Dresden, Germany

**Keywords:** Bacterial contamination, *Clostridium perfringens*, Embalming, Fixation, Gross anatomy teaching

## Abstract

Post-mortem specimens used for anatomy teaching are commonly embalmed using compositions of chemicals, with the objective to maintain tissue quality and to avoid putrefaction. Monitoring for bacterial or fungal contamination is becoming increasingly important especially when measures are taken to minimize exposure by chemicals such as formaldehyde. In this case, random swabs were taken from six corpses embalmed with ethanol-glycerin and Thiel embalming. Cultures and MALDI-TOF analyses yielded four cases of *Clostridium perfringens* contamination. *C. perfringens* is of special interest as a human pathogen. A potential source was identified in the containers filled with the moistening solution. Cross contamination with *Clostridium* species has likely occurred between corpses sharing the moistening solution and soaking the cover linen directly within the containers. To minimize any risk for those exposed, the moistening solutions were discarded and all equipment thoroughly disinfected. The specimens had to be cremated as they formed a potential source of *Clostridium* spores. Deviating from previous routines it was formalized that the cover linen must not be submerged in the moistening contains rather than moistening the specimens directly with dedicated vessels. Follow-up analyses yielded no further contamination with *C. perfringens*.

## Introduction

Human post-mortem tissues provide a valuable resource for undergraduate and professional teaching. Commonly, these tissues are embalmed using different chemicals, primarily with the objective to maintain tissue quality and to avoid putrefaction (Hammer et al. 2015; Thiel 1992). A recent study has questioned the disinfecting effects of commonly used embalming solutions (Balta et al. 2019). So far, little research has been conducted on the microbiota that may survive the embalming process, or even may cause cross contamination of other corpses (Brenner 2014). Bacterial or fungal contamination of anatomical specimens with human pathogens may pose staff and students likewise at risk. In particular, sporulating bacteria may be relevant owing their ability to survive in hostile environments for longer durations (Horneck et al. 1994). *Clostridium* species such as *Clostridium perfringens* may lead to the development of gas gangrene, potentially with fatal outcome (Suerbaum et al. 2020). *C. perfringens* is known to be resistant even to 50% ethanol environments for more than one hour (Koransky et al. 1978). For reasons of hygiene and workplace safety, it is important to understand which microorganisms may find a suitable environment to grow on chemically embalmed specimens, and which ones may be a potential harm to living humans. We report on an incidental finding of *C. perfringens* species on ethanol–glycerin (Hammer et al. 2012) and Thiel (Thiel 1992, 2002) embalmed specimens and which measures were taken to minimize the risk for students and staff in anatomy.

## Materials and methods

### Sampling and testing site

Samples were collected from six post-mortem corpses using a sterile bacterial swab and then hydrated in 1 ml Amies-Media (eSwab, Hain Lifescience, Nehren, Germany). Three were embalmed with ethanol-glycerin (Hammer et al. 2012) and three with Thiel solution (Thiel 1992, 2002). Sampling took place at the beginning of the student dissection course. A multi-site probe acquisition was performed from the nasopharynx, oral cavity and anogenital region. For the moistening solution containers, sterile 10-ml sample tubes (Süsse Labortechnik GmbH & Co. KG, Gudensberg, Germany) were used. Students were wearing two pairs of clinical gloves, clean lab coats (as always) and FFP2 masks (due to the COVID-19 pandemic). Donations of post mortem-tissues for research and teaching purposes at the Medical University of Graz (Austria) are regulated under the Styrian death and funeral act. The swab samples obtained as part of this report have been retrieved for quality assurance purposes in teaching and prosection maintenance, hence no ethical approval was deemed necessary.

### Incubation and bacterial species identification

The swabs were streaked out on agar plates (PVX, Columbia, MCConkey and Schaedler; BD, Heidelberg, Germany) and then incubated under anaerobic (for Schaedler) and aerobic (others) conditions at 37 °C for 24 h (Suerbaum et al. 2020). The samples retrieved from storage containers with the moistening chemicals were sterile filtered under vacuum and the filters then incubated on Schaedler agar at 37 °C for 48 h under aerobic and anaerobic conditions. To make sure that proper growth of each detected bacterium was achieved, all detected colonies were then applied to Columbia agar (aerobic conditions) and/or Schaedler agar (anaerobic conditions) with incubation at 37 ± 2 °C for 48 h.

Bacterial identification was performed by separating single colonies onto new agar plates, and then analyzed by Maldi-TOF MS (Vitek MS, bioMérieux SA, Marcy l’Étoile, France). One or two colonies of each isolate were directly spotted on the manufacturer’s proprietary sample plates following the manufacturer`s protocols and recommendations. A 1- µL volume of CHCA matrix solution (α-cyano-4-hydroxycinnamic acid; bioMérieux Inc.) was then applied to each sample and air-dried for 5 min at room temperature for crystallization. For species identification of each isolate, a total of four spots were analyzed on the VITEK MS system. The MALDI-TOF MS instrument used in this study was equipped with a 337 nm-fixed focus nitrogen laser of 50 Hz frequency, the software program was the VITEK MS IVD analysis software version 3.2. *Escherichia coli* ATCC 8739 was used as positive control.

## Results

Bacteria species were detected on five of the six corpses investigated. The resulting bacterial cultures yielded four cases of *C. perfringens*, two *Propionibacterium* species, and one case each of *C. innocuum*, *Escherichia coli* and *C. tertium*, respectively. Four of the five containers with the moistening solution were contaminated with the bacterial species detected: *C. perfringens* and *C. paraputrificum*, *Enterococcus faecalis*, *Enterococcus faecium* and *Leuconostoc mesenteroides* (Table 1). These observations were made irrespective of the type of moistening solution.

**Table 1:** List of corpuses and tons and the respective found bacterial species

Corpse no.	Bacterial species	Ton number	Bacterial species
K104	*Clostridium innocuum*, *Propionibacterium spp.*	1 (Thymol based)	*Clostridium perfringens*, *Clostridium paraputrificum*
K108	*Clostridium perfringens*	2 (Thymol based)	Sterile
K110	*Clostridium perfringens*, *Escherichia coli*	3 (Thiel-solution)	*Enterococcus faecium*, *Enterococcus faecalis*, *Leuconostoc mesenteroides*
K111	*Clostridium perfringens*	4 (Thiel-solution)	*Clostridium perfringens*, *Enterococcus faecalis*
K121	*Clostridium perfringens*, * Clostridium tertium*, *Propionibacterium spp.*	5 (Thiel-solution)	*Clostridium perfringens*, *Clostridium paraputrificum*
K125	No growth		

## Discussion

Prior to the anatomical dissection of human corpses, two main steps of embalming are commonly performed: chemical fixation and conservation. Anatomical fixation relates to the procedures to interfere tissue decay and to prevent from (further) contamination and autolysis. Chemicals used for anatomical conservation help to maintain the state of fixation (Hammer et al. 2012). For safety reasons it is vital to evaluate the risk of bacterial/fungal contamination for both infection containment and hygiene purposes to work safely with embalmed corpses. Compared to formaldehyde based embalming which showed high bactericidal effects over 8 months of investigation in previous studies (Balta et al. 2019), Thiel solution and ethanol–glycerin embalming methods could be considered mild embalming methods with low formaldehyde concentrations aiming at good preservation of tissue softness for surgical training (Brenner 2014). Therefore, ongoing microbiological testing for quality assurance is performed. From the list of bacteria observed here, *C. perfringens* seems of special interest. The germ is widely distributed in soil and is regularly found in the human gastrointestinal tract without causing symptoms (Ohtani and Shimizu 2016). As a human pathogen, it is one of the major causes for gas gangrene. Less frequently gas gangrene is caused by *C. septicum*, *C. histolyticum* and more recently *C. sordellii* (Buboltz and Murphy-Lavoie 2022). For *C. tertium*, *C. paraputrificum* and *C. innocuum*, as found in the samples reported in this study, only a few case reports, including abscess formation, bacteremia, diarrhea and only one case of gas gangrene were  published (Fujitani et al. 2007; Vijayvargiya et al. 2020; Chen et al. 2022).

Though infection following skin injury hardly ever happens in healthy individuals. The inoculation of *C. perfringens* may prove detrimental in the presence of immunosuppression (Leiblein et al. 2020). Hence, special care must be taken to minimize any risk of infection (Suerbaum et al. 2020). Though cases of *Clostridium* species infection seem low in general (incidence of 1.8 per 100.000 persons per year), 42% of *Clostridium* infections are caused by *C. perfringens* (Fukui et al. 2017). Previous studies have yielded that *Clostridium* species are capable of surviving ethanol concentrations of up to 50% for more than one hour of incubation (Koransky et al. 1978).

Based on the observation of the bacteria above, three potential explanations were found for the onset of *Clostridium* species: 1) ubiquitarian environmental contamination, 2) contamination from storage systems or applicants, or 3) cross contamination from corpses of body donors harboring *C. perfringens* in their gastrointestinal tract. The presence of *C. perfringens* on the corpses and moistening solutions led to the conclusion that cross contamination seems to be the most likely explanation. We here hypothesize that a contamination of one (or several corpses) was spread when soaking the cover linen by submersion in the same moistening solution.

### Measures

To minimize the risk of infection for those working with the tissues, the following measures were taken: The container moistening solutions were  replaced followed by thorough disinfection of all tables, containers and other dissection room equipment (disinfectant used: NOWA ISR 700, Tana, Mainz, Germany). Further to this, it was formalized as a standard procedure that the linen used to cover the corpses must not be submerged in the moistening contains rather than pouring the moistening fluid directly onto the linen on top of the corpses, using dedicated vessels. Follow-up analyses yielded no further onset of *C. perfringens* contamination in any of the tissues nor in the containers for the moistening solution.

## Data Availability

Data will be made available upon reasonable request.
