# Publisher Correction: Incidental finding of *Clostridium perfringens* on human corpses used for the anatomy course

**DOI:** 10.1007/s12565-023-00704-y

**Published:** 2023-01-31

**Authors:** Alexander M. Kerner, Andrea J. Grisold, Freyja-Maria Smolle-Jüttner, Niels Hammer

**Affiliations:** 1 Division of Macroscopic and Clinical Anatomy, Gottfried Schatz Research Center, Medical University of Graz, Auenbruggerplatz 25, 8036 Graz, Austria; 2 D & R Institute of Hygiene, Microbiology and Environmental Medicine, Medical University of Graz, Neue Stiftingtalstrasse 6, 8010 Graz, Austria; 3 Division of Thoracic and Hyperbaric Surgery, Department of Surgery, Medical University of Graz, Auenbruggerplatz 29, 8036 Graz, Austria; 4 Department of Orthopedic and Trauma Surgery, University of Leipzig, Leipzig, Germany; 5 Division of Biomechatronics, Fraunhofer Institute for Forming Tools, Dresden, Germany

**Publisher Correction: Anatomical Science International (2022) 98:151–154** 10.1007/s12565-022-00699-y

In the original publication of the article, the affiliation 1 has been duplicated as affiliation 6 during the production process. The publisher apologizes for the inconvenience caused.

The original article has been corrected.

